# Mental imagery modulates bistable perception in a modality-specific manner

**DOI:** 10.1038/s41598-026-44578-2

**Published:** 2026-03-19

**Authors:** Luca Verebélyi, Ágnes Welker, Kökény Kovács-Deák, Ferenc Gombos, Gábor Péter Háden, István Winkler, Ilona Kovács

**Affiliations:** 1 HUN-REN Research Centre for Natural Sciences, Budapest, Hungary; 2 Semmelweis University Doctoral School, Budapest, Hungary; 3 Pázmány Péter Catholic University, Budapest, Hungary; 4 HUN-REN-ELTE-PPKE Adolescent Development Research Group, Budapest, Hungary; 5 Faculty of Education and Psychology, Eötvös Loránd University, Budapest, Hungary

**Keywords:** Mental imagery, Bistable perception, Binocular rivalry, Auditory streaming, Imagery-based priming, Multisensory imagery, Neuroscience, Psychology, Psychology

## Abstract

Multimodal approaches are essential for understanding the mechanisms and constraints of mental imagery, yet few studies have directly compared its perceptual effects across sensory systems. This study explored how mental imagery influences bistable perception in vision and audition by comparing imagery-based and sensory-based priming in binocular rivalry and auditory streaming. In auditory streaming, an acoustic bias promoting segregation increased the likelihood of an early segregated percept, whereas an imagery priming did not. Binocular rivalry exhibited robust and asymmetric priming effects within the first few seconds, with both imagery and physical cues biasing perceptual onset. While both physical and imagery priming significantly influenced the initial percept in binocular rivalry, only physical priming affected perceptual reports in auditory streaming. Notably, the strength of imagery priming in vision correlated with self-reported imagery vividness, supporting the functional relevance of individual differences in mental imagery. These findings indicate that imagery can bias visual perception but may have limited influence in audition under current task constraints, likely due to modality-specific dynamics and early integration biases. This study provides behavioural evidence for the modality-specific impact of mental imagery and underscores the value of cross-modal designs in understanding how internal representations shape conscious perception.

**Supplementary Information:**

The online version contains supplementary material available at 10.1038/s41598-026-44578-2.

## Introduction

As a form of cognitive simulation, mental imagery provides a unique opportunity to study the contents and mechanisms of conscious experience in the absence of direct sensory input. While research on mental imagery has historically focused on a single modality – most often vision – conscious experience itself is inherently multisensory, drawing upon integrated information from across the senses rather than isolated, unisensory inputs. Multisensory integration is a defining feature of perception and cognition, and growing evidence suggests that this integrative structure extends to mental imagery as well. People can vividly imagine not only what they see, but also what they hear, feel, taste, or smell, and these imagined experiences often interact across modalities^1,2^. Despite this, empirical research on mental imagery has focused predominantly on the visual domain, leaving auditory, tactile, and other forms of imagery comparatively understudied^3,4^.

If conscious experience is inherently multisensory, then a comprehensive account of its neural and cognitive underpinnings must consider imagery across multiple modalities. Understanding how different forms of imagery arise, how they vary in vividness, and how they interact across sensory systems may offer unique insights into the architecture of conscious experience. To advance this goal, the present study introduces a modality-general experimental framework for assessing imagery in both the auditory and visual domains. This common paradigm allows us to gauge imagery in the two modalities under comparable conditions.

A variety of objective methods have been employed to assess mental imagery, including fMRI^5^, EEG^6^, galvanic skin response^7^, eye-tracking^8^, and particularly binocular rivalry^9^. Findings on the use of binocular rivalry to assess mental imagery remain inconsistent. While some studies report that self-rated vividness modulates perceptual dominance^9–12^, others find no such link^13,14^. Importantly, Bouyer et al. (2025)^14^ reported a correlation between VVIQ and binocular rivalry, and found that imagery priming scaled with trial-level vividness ratings; however, they did not observe a correlation between mean vividness ratings or VVIQ scores and priming values. These mixed results challenge the reliability of binocular rivalry as an objective marker of imagery strength and fuel ongoing debates in the field. Notably, the more objective no-report variants of binocular rivalry, which eliminate introspective bias by relying on behavioral or physiological indicators of percept dominance, have been used only sparingly^15^. Furthermore, although visual imagery has been extensively studied in binocular rivalry paradigms, equivalent objective methods have not yet been established for auditory imagery, highlighting a significant gap in the field.

Bi-/multistable perception^16^ offers a powerful window into the dynamics of conscious experience, as it involves perceptual alternations despite constant sensory input. In such paradigms, the external stimulus remains unchanged, yet the perceptual interpretation shifts over time, making them ideal for isolating neural and cognitive correlates of conscious awareness^17,18^. Binocular rivalry, in particular, has been widely used to study visual awareness: when each eye is presented with a different visual stimulus, perception spontaneously alternates between the two competing images^16,19,20^. A parallel phenomenon exists in the auditory domain in the form of auditory streaming^21,22^, where sequences of tones at different frequencies can be perceived either as an integrated whole or as segregated streams, with perception fluctuating over time^23–26^.

In our modality-general framework we combined bistable stimulation in both modalities with imagery priming. Perceptual priming – where prior exposure to a stimulus biases subsequent perception – has long been used to study the pre-activation of sensory and conceptual representations^27,28^. In the context of bistable perception, priming with low-level cues can shift the likelihood of one perceptual interpretation over another, even when the physical stimulus remains ambiguous^22,29,30^, and this has also been suggested to work using imagery-based cues^9,15^. In the present study, we apply imagery priming in both modalities, allowing us to compare how imagined content shapes the dynamics of bistable perception across sensory systems.

**Fig. 1:**
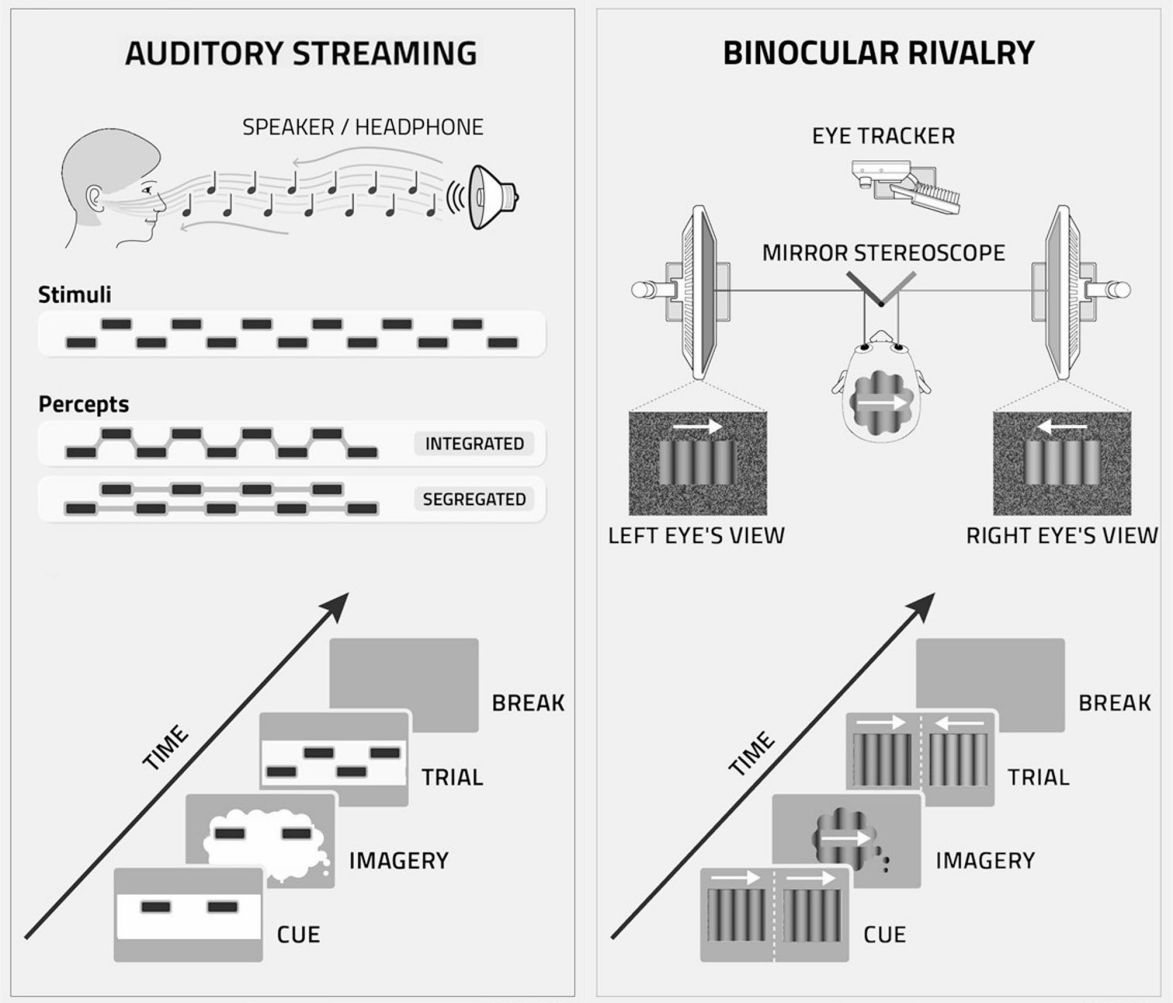
Imagery priming in two perceptual modalities. In Auditory Streaming priming, stimuli consist of bistable streams of sounds at two distinct frequencies. These were perceived either as a single integrated sound sequence composed of the two tones with different frequencies or as two segregated streams, each consisting of tones of only one of the the two frequencies. In imagery priming, participants first listened to a sequence of one of the two tones from the ambiguous sequence serving as a cue for subsequent imagination. After imagining the cued stream, the ambiguous stimulus was presented, and participants reported whether they perceived it as being integrated or segregated. Modulation of the perceptual reports of the ambiguous stimulus due to imagery priming was assessed in the analyses. In Binocular Rivalry priming, stimuli were presented through a stereoscope, with each eye receiving sinusoidal contrast grating patterns moving in opposite directions. This bistable stimulus elicited perceptual alternations between the two motion directions. An eye-tracker recorded the direction of the optokinetic-nystagmus reflex, providing an implicit, no-report measure of perceived motion. In imagery priming, participants first viewed an unambiguous motion stimulus that cued subsequent imagination. After imaging the cued direction, rivalrous gratings were presented. Modulation of the eye-movement pattern due to imagery priming was assessed in the analyses.

To assess auditory imagery, we employed an adaptation of the auditory streaming paradigm^31,32^. Auditory perception involves the organization of sound into coherent perceptual streams^22,33^. When a sound sequence is formed from two tones with similar acoustic features – such as frequency, amplitude, harmonics, or pitch^34^– they are typically perceived as a single, integrated auditory stream. In contrast, if the tones differ significantly in these characteristics, they are perceived as segregated, independent streams (see Fig. 1.). Once participants were able to distinguish between segregated and integrated percepts of the sounds in the ambiguous tone sequence, two types of priming conditions were introduced: physical priming previously documented in the literature^35,36^, and imagery-based priming, developed by our research group (see Fig. 1. and Methods). In the physical priming condition, shortly before the ambiguous sound sequence, participants were presented with a repetitive sequence of one of the two sounds from the ambiguous sequence (i.e., one of the would-be streams if the ambiguous sequence was perceived as segregated), because such priming is known to promote stream segregation^35,36^. In the imagery-based priming condition, participants were instructed to imagine the repeating sound between the biasing sequence and the ambiguous sequence.

To assess visual imagery vividness, we used a no-report binocular rivalry paradigm^15,37,38^. Participants viewed two gratings moving in opposite directions – one presented to each eye – through a mirror stereoscope (see Fig. 1.). This setup induced spontaneous alternations in perceptual dominance. Perceptual changes during rivalry, including spontaneous reversals, are closely tracked by the emergence of saccadic eye movements associated with optokinetic nystagmus, which reliably reflect the currently dominant percept. The optokinetic nystagmus reflex is triggered only by actual visual input and not by imagined motion, making it a robust objective measure of perceptual dominance without requiring subjective reports. These eye movements were recorded with an eye-tracking device to capture perceptual dominance objectively^15,38,39^. Because optokinetic nystagmus reflects what participants actually perceive without requiring any button press or verbal report, this method minimizes potential interference from higher-level cognitive processes^37^. We also used two types of priming in Binocular Rivalry. In the physical priming trials, an unambiguously moving grating was shown to bias perception. In the imagery priming trials, a short unambiguous cue was presented to cue imagery.

The above design allowed us to directly compare the effects of external sensory input and internal mental imagery on perceptual dominance during binocular rivalry. As physical priming has been shown to be effective in both auditory streaming^40^ and binocular rivalry^11,13^, we expected to replicate these effects in our experiments. While imagery-based priming has only been demonstrated in binocular rivalry^9–12^, we hypothesized that it would also influence perception in the auditory domain. This expectation was based on the structural parallels between the designs across the two modalities, and the reported effectiveness of physical priming in both cases.

In our analysis, first we compared the time course of priming effects in auditory streaming and binocular rivalry priming, and we expected to see modality differences in the dynamics of perceptual competition. Binocular rivalry is governed by reciprocal inhibition between monocular neurons and higher-level visual patterns; it often shows rapid alternations and can be sensitive to subtle biases^19^. Auditory streaming, in contrast, may involve slower build-up of perceptual organisation and different forms of neural adaptation^22^. By comparing how long imagery-induced biases last in each modality (e.g., how quickly they decay), we tested whether the temporal stability of imagery effects is shaped by the modality-specific architecture of bistability. ​​Comparing the time course of priming could also inform us whether visual and auditory imagery are maintained via similar memory systems (e.g., visual working memory vs. echoic/auditory working memory), or whether they differ in temporal precision and persistence. If both modalities show similar decay rates or time-dependent effects, this could suggest a common mechanism for imagery-based modulation (e.g., attentional or predictive processes). If they differ, it may point to separate imagery-perception coupling mechanisms, as was shown for auditory and visual multistability^41^. This informs us whether conscious modulation by imagery is a modality-general cognitive function (e.g., via frontoparietal networks) or modality-specific.

We then investigated whether the types and durations of percepts were modulated by priming. If mental imagery influences the competition between perceptual interpretations, we would expect it not only to bias the initial percept (i.e., what is seen or heard first), but also to alter the relative dominance and stability of each percept over time. Specifically, imagery congruent with one interpretation may lead to more frequent or longer-lasting occurrences of that percept.

Finally, we analysed whether individual differences in self-reported imagery vividness correlated with the strength of imagery priming. If vivid mental imagery more strongly engages sensory representations, participants reporting more vivid imagery should show stronger perceptual modulation in response to imagery cues. Such a relationship would support the idea that imagery vividness reflects functionally relevant differences in top-down sensory activation, rather than being purely subjective or metacognitive. It also contributes to broader debates about whether imagery vividness is a trait-like predictor of perceptual influence, and whether behavioural measures of imagery effects can serve as objective correlates of subjective vividness.

## Results

### Time course of priming effects in auditory streaming and binocular rivalry priming

**Fig. 2:**
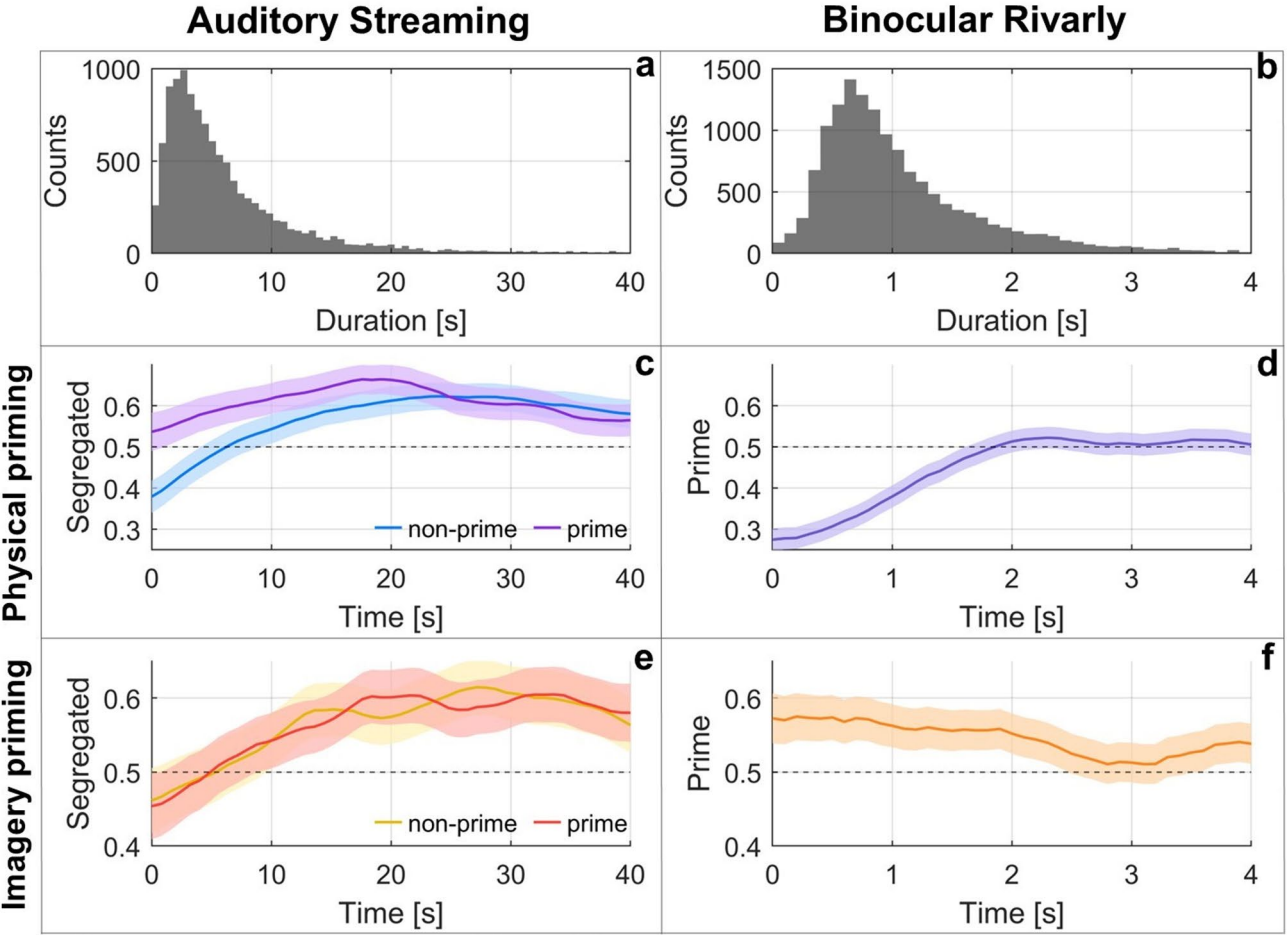
Time course of physical and imagery priming effects in Auditory Streaming and Binocular Rivalry. Left column (**a**, **c**, **e**,): Auditory Streaming; right column (**b**, **d**, **f**): Binocular Rivalry. **a-b**: Histograms of dominant percept durations. **c-f**: Time course of priming effects analyzed with sliding windows (Auditory Streaming: 9-second window, 7.2-second overlap; Binocular Rivalry: 1-second window, 0.9-second overlap) Shaded area: 95% confidence interval. Proportions calculated from dominant (integrated or segregated, right or left) percepts within each window.; **c**, : proportion of segregated percepts in the baseline (non-prime) and physical priming (prime) condition **d**: proportion of prime-congruent percepts in physical priming **e**: proportion of segregated percepts in the physical prime with extended delay (non-prime) and the imagery priming (prime) condition;**f**: proportion of prime-congruent percepts in imagery priming.

We analyzed the durations of dominant percepts in both paradigms and visualized them using histograms (Fig. 2a-b). In Auditory Streaming, a total of 11,580 dominant segments (either integrated or segregated) were identified across all trials, with an average duration of 7.38 s and a median of 4.57 s. The Binocular Rivalry yielded 14,205 dominant segments (left or right), with an average duration of 1.11 s and a median of 0.88 s.

To examine temporal dynamics, we applied a sliding window analysis averaging across trials within each condition (Fig. 2c-f). Window size was set close to the average dominant percept duration: 9 s with 7.2 s overlap for Auditory Streaming, and 1 s with 0.9 s overlap for Binocular Rivalry. Panels c-f display the proportion of prime-congruent percepts (i.e., percepts consistent with the direction or type of the preceding prime stimulus), separately for the two modalities and the two types of priming (physical, imagery). For Auditory Streaming this means the segregated percept, because the bias promoted segregation, which was compared to the proportion of the same percept without priming (i.e., the baseline and the extended delay condition for physical and the imagery priming, respectively). In Binocular Rivalry (Fig. 2d, f), deviations from 0.5 indicate a priming effect; in Auditory Streaming, differences between the two comparable conditions (baseline vs. physical priming and physical priming with extended delay vs. imagery priming) indicate a priming effect.

The Auditory Streaming paradigm is typically characterized by a strong integrated bias^42–44^, so we set the parameters to ensure segregated perception would predominate after the initial bias, as evidenced by the results. In the case of physical priming, the proportion of segregated perceptions increased during the first approximately 15 s relative to baseline. In the case of imaginary priming, we see no difference between the physical priming with extended delay and the imaginary priming condition.

In Binocular Rivalry, we found a negative effect of physical priming (Fig. 2df) and a positive effect of imagery priming (Fig. 2f) on the proportion of prime-congruent percepts, as reflected in the deviations from 0.5 shown in the figures.

### Types of first percepts

We analyzed the types of first dominant percepts as a function of priming across trials – priming-congruent (segregated) vs. priming-incongruent (integrated) in Auditory Streaming, and leftward vs. rightward in the Binocular Rivalry – computing contingency tables and chi-square tests separately for each paradigm and priming condition. Results are presented in Table 1.

In Auditory Streaming, significant association was found between priming and initial percept type in the physical (χ²(1, *N* = 676) = 21.33, *p* <.001, Cramer’s V = 0.178, BF₁₀ = 3737) but not in the imagery priming condition (χ²(1, *N* = 608) = 0.119, *p* =.730, Cramer’s V = 0.014, BF₁₀ = 0.103).

In contrast, in Binocular Rivalry, a significant association was observed between prime type and initial percept type for both the physical (χ²(1, *N* = 1215) = 137.088, *p* >.001, Cramer’s V = 0.335, BF₁₀ = 3.492*1028) and the imagery condition (χ²(1, *N* = 1227) = 19.189, *p* >.001, Cramer’s V = 0.125, BF₁₀ = 1062.612).

**Table 1:** Types of first percepts** (a)** Contingency table **(b)** Chi-square analysis.

Auditory Streaming					Binocular Rivalry					
	Type of First Percept				Type of First Percept					
Condition		Integrated	Segregated	Total	Condition		Right	Left	Total	
Baseline	Count	280	100	380	Left Physical Priming	Count	374	114	488	
	%	74%	26%	100%		%	77%	23%	100%	
Physical Priming	Count	168	128	296	Right Physical Priming	Count	315	412	727	
	%	57%	43%	100%		%	43%	57%	100%	
Physical Priming with extended delay	Count	194	114	308	Left Imagery Priming	Count	222	264	486	
	%	63%	37%	100%		%	46%	54%	100%	
Imagery Priming	Count	193	107	300	Right Imagery Priming	Count	433	308	741	
	%	64%	36%	100%		%	58%	42%	100%	
	Auditory Streaming					Binocular Rivalry				
Condition	Value	df	p	Cramer’s V	BF₁₀	Value	df	p	Cramer’s V	BF₁₀
Physical Priming *Χ²*	*21.33*	*1*	*< 0.001*	*0.178*	*3737*	137.088	1	< 0.001	0.335	3.492*10^28^
Imagery Priming *Χ²*	*0.119*	*1*	*0.730*	*0.014*	*0.103*	19.189	1	< 0.001	0.125	1062.612

### Lengths of first percepts

We examined the duration of first dominant percepts after the priming in both Auditory Streaming (first reported percept) and Binocular Rivalry (first leftward or rightward percept after the short mixed period – see Methods).

**Fig. 3:**
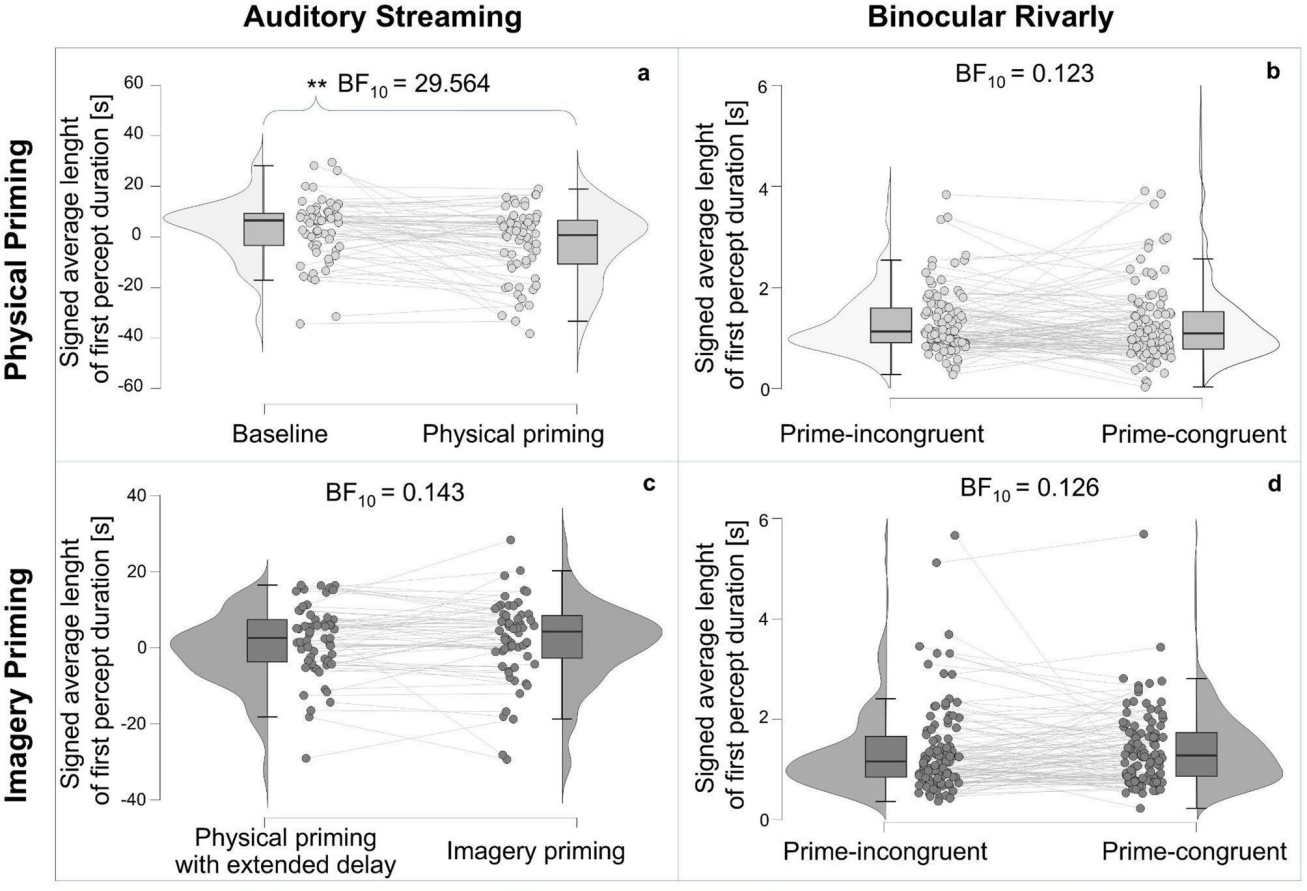
Aggregated results of the lengths of the first percepts in Auditory Streaming and Binocular Rivalry. Individual data points are represented by dots, while box plots indicate the medians and interquartile ranges. Violin plots further illustrate the underlying data distribution. All figures present results from Bayesian non-parametric analyses. **(a)** Comparison of the signed average durations of first percepts in the baseline and the physical priming condition of the auditory streaming paradigm. **(b)** Mean duration of prime-congruent and prime-incongruent first percepts under the physical priming condition in the binocular rivalry paradigm. **(c)** Comparison of the durations of the signed average length of first percepts under physical priming with extended delay and imagery priming condition in the auditory streaming paradigm. **(d)** Mean duration of prime-incongruent and prime-congruent first percepts under the imagery priming condition in the binocular rivalry paradigm.

Since there is an initial bias toward integrated percepts in Auditory Streaming (Table 1; Fig. 2c 2e), we calculated the average signed length of the first percept in each condition and subject, assigning the segregated percept a negative sign and the integrated percept a positive sign. Since data were not normally distributed (Shapiro–Wilk test: *p* <.05 across all conditions) paired Wilcoxon test were used. We found that physical priming (Mdn = 712 ms; M = −2723 ms) induced a significant difference (*p* =.002, BF₁₀ = 29.564) compared to the baseline (Mdn = 6517 ms; M = 3239 ms) in the average signed length of the first percept (Fig. 3a) while imagery priming (Mdn = 4144; M = 2017 ms) did not (*p* =.944, BF₁₀ = 0.143) significantly differ from physical priming with extended delay (Mdn = 2463 ms; M = 1809 ms) with moderate evidence for H₀ (Fig. 3c).

In Binocular Rivalry, multiple trials were available in each condition, allowing within-subject averaging of prime-congruent and prime-incongruent first percept durations. Since data were not normally distributed (Shapiro–Wilk test: *p* <.001 across all conditions), paired Wilcoxon tests were used. No significant difference was observed in the physical priming condition between prime-congruent (Mdn = 1103 ms, M = 1369 ms) and prime-incongruent (Mdn = 1130 ms, M = 1330 ms) first percepts durations (*p* =.372, BF₁₀ = 0.123; Fig. 3b). Likewise, in the imagery priming condition, durations of prime-congruent (Mdn = 1296 ms, M = 1419 ms) and prime-incongruent (Mdn = 1149 ms, M = 1395 ms) first percepts did not differ (*p* =.344, BF₁₀ = 0.126; Fig. 3d). Both comparisons yielded moderate evidence for the null hypothesis.

### Correlation of self-reported vividness with imagery priming

**Fig. 4:**
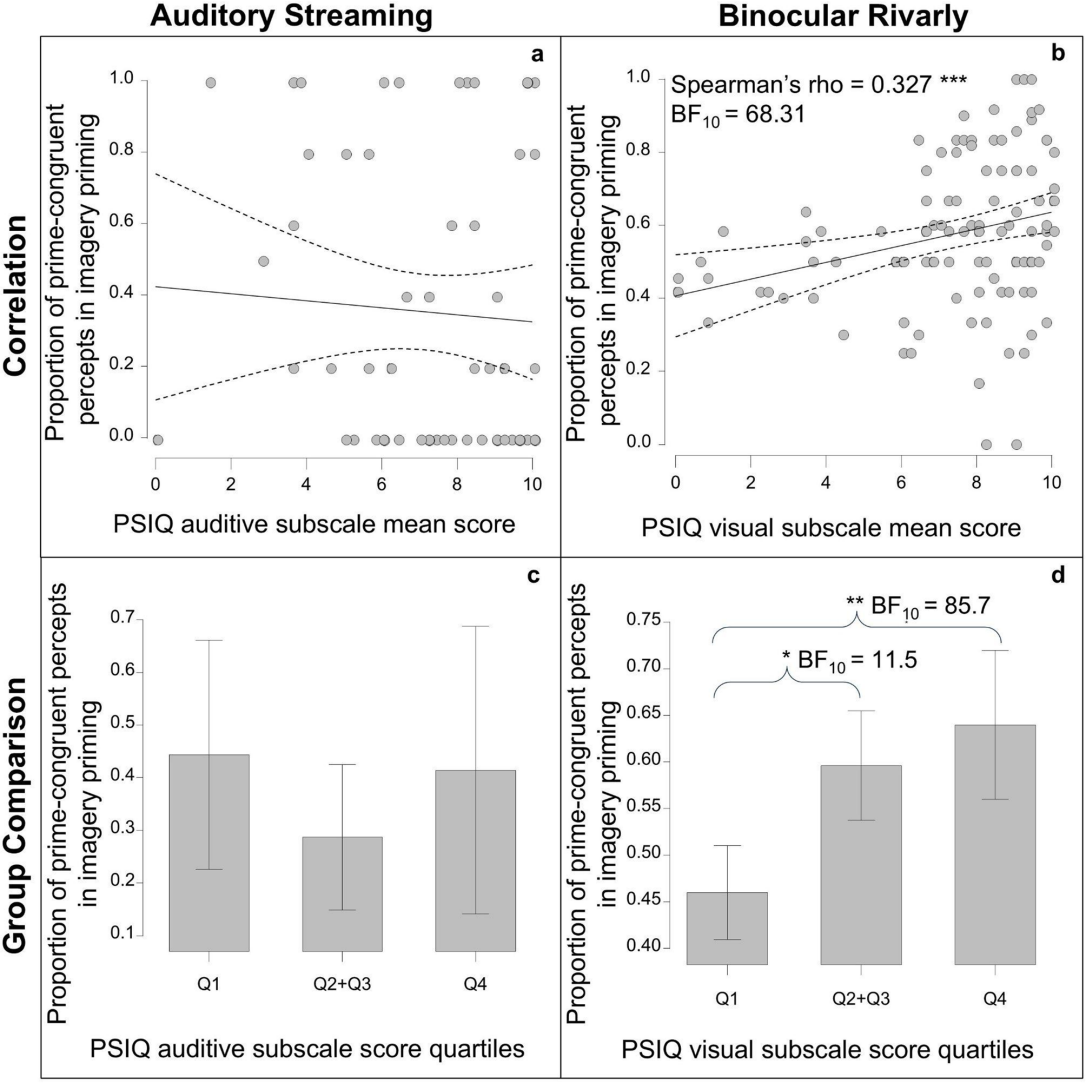
Imagery Priming **a**,** b**: Correlation between PSIQ auditive (**a**) and visual (**b**) subscale scores and the proportion of prime-congruent first percepts during imagery priming. The Dashed lines represent the 95% confidence interval. **c**,** d**: Mean proportion of prime-congruent first percepts in the imagery priming conditions across the PSIQ auditive (**c**) and visual (**d**) imagery groups (Q1, Q2 + Q3, Q4; the lowest quartile, the two middle quartiles pooled, and the highest quartile, respectively), with error bars indicating 95% confidence intervals; asterisks and Bayes Factors (BFs) indicate results from ANOVA and Bayesian ANOVA. (* *p*<.05; ** *p*<.01; *** *p*<.001).

Imagery priming was observed only in Binocular Rivalry, specifically in the type of the initial percepts, and thus further analyses focused on this condition. For each participant, we calculated the proportion of prime-congruent first percepts and examined its association with the PSIQ visual subscale. The PSIQ visual scores were not normally distributed (Shapiro–Wilk *p* <.001), whereas the proportion of prime-congruent first percepts was (Shapiro–Wilk *p* =.072), therefore, a non-parametric correlation was applied. Spearman’s rank correlation revealed a positive, moderate relationship between the PSIQ visual subscale and the proportion of prime-congruent first percepts (ρ = 0.327, *p* <.001). The effect size based on Fisher’s *z* was 0.339, indicating a moderate effect. Bayesian Kendall’s tau (τ = 0.233) also provided strong evidence for this association (BF₁₀ = 68.312; Fig. 4b).

Parallely, in Auditory Streaming the ratio of prime-congruent first percepts and its association with the PSIQ auditive subscale was calculated for each participant. As the PSIQ auditive subscale score significantly deviated from normal distribution (Shapiro–Wilk *p* <.001), non-parametric correlation was employed. Spearman’s rank correlation (*p* =.647), and its effect size (Fisher’s *z* was − 0.060) indicated no significant association. Bayesian Kendall’s tau did not either support evidence for a significant association (BF₁₀ = 0.200; Fig. 4a).

To investigate group-level differences, participants were divided into 3 groups based on their PSIQ scores: Q1, the lowest quartile, Q2 + Q3 the two middle quartiles, and Q4, the highest quartile (Binocular Rivalry: Q1: PSIQ visual score < 6.55, *n* = 27; Q2 + Q3: 6.55 ≤ PSIQ visual score < 9.20, *n* = 53; Q4: 9.20 ≤ PSIQ visual score, *n* = 28; Auditory Streaming: Q1: PSIQ auditory score < 5.85, *n* = 16; Q2 + Q3: 5.85 < = PSIQ auditory score < 7.8, *n* = 30; Q4: 7.8 < = PSIQ auditory score, *n* = 16). In Binocular Rivalry an ANOVA on the proportion of prime-congruent first percepts in the Imagery Priming condition was conducted. As the assumption of homogeneity of variances was violated (Levene’s test: *p* =.011), a Welch correction was applied, revealing a significant group effect (*p* <.001; BF_M_ = 18.812). Tukey post-hoc tests showed that participants in Q4 (highest imagery) reported significantly more prime-congruent first percepts than those in Q1 (*p* =.004; Cohen’s *d* = 0.936; BF₁₀ = 85.725), and Q2 also differed significantly from Q1 (*p* =.005; Cohen’s *d* = 0.954; BF₁₀ = 835.755). No significant differences were found between Q3 and any other group (Q1: *p* =.240, BF₁₀ = 1.038; Q2: *p* =.368, BF₁₀ = 0.683; Q4: *p* =.368, BF₁₀ = 0.580), nor between Q2 and Q4 (*p* = 1.000; BF₁₀ = 0.279) (Fig. 4b).

In a follow-up analysis, we combined the middle quartiles (Q2 + Q3) and repeated the ANOVA. The assumption of homogeneity of variances was acceptable (Levene’s test: *p* =.053). The analysis indicated a significant group difference (*p* =.002; BF_M_ = 15.719). Tukey post-hoc comparisons showed that Q1 had significantly lower prime-congruent perception rates than both Q2 + Q3 (*p* =.010; Cohen’s *d* = 0.702; BF₁₀ = 11.522) and Q4 (*p* =.002; Cohen’s *d* = 0.928; BF₁₀ = 85.726), while Q2 + Q3 and Q4 did not differ significantly (*p* =.599; BF₁₀ = 0.339) (Fig. 4d). In Auditory Streaming, the assumption of normality was violated (Shapiro-Wilk, *p* <.001), so a Kruskal-Wallis test was performed, which showed no significant difference between the different imagery groups (*p* =.521, BF₁₀ = 0.295) (Fig. 4c).

## Discussion

In this study, we implemented a modality-general design to directly compare the influence of external sensory input and internally generated mental imagery on perceptual dominance across two bistable paradigms: auditory streaming and binocular rivalry. Building on previous findings that physical priming modulates perception in both modalities^9,10,15,35,36^, we first sought to replicate these effects using unambiguous sensory cues. Extending this logic, we tested whether imagery-based priming, previously demonstrated only in the context of binocular rivalry^9–12,15^, would similarly modulate perception in the auditory domain. This hypothesis was motivated by the structural similarity of the two paradigms and the established efficacy of physical priming across modalities.

We first compared the time course of priming effects across modalities, anticipating differences in the dynamics of perceptual competition. Binocular rivalry typically involves rapid perceptual alternations and is sensitive to subtle biases^19^, whereas auditory streaming is characterised by a slower build-up of perceptual organisation^22^. By examining the duration and decay of imagery-induced biases, we tested whether the temporal stability of priming effects reflects modality-specific properties of bistability. In auditory streaming, an initial bias toward the integrated percept was observed, supporting the classical integration-first build-up theory^42–44^. However, segregated physical priming produced a significant association between prime type and the identity of the first percept. This indicates that an externally presented cues can bias early perceptual organization toward segregation in the auditory domain^32,40,45^ despite a default bias toward integration. Notably, this effect was restricted to physical priming; imagery priming did not significantly influence the type of the initial percept. Thus, while bottom-up cues successfully altered perceptual selection, internally generated auditory imagery did not measurably bias initial perceptual choice under the present conditions.

On the other hand, binocular rivalry revealed distinct and asymmetric priming effects. Physical priming exerted a negative effect on the proportion of prime-congruent percepts, reflected in deviations below the 0.5 baseline. In contrast, imagery priming showed a positive effect, increasing the proportion of prime-congruent percepts above chance level (0.5).

When combined, these effects yielded a modest increase in prime-congruent percepts during the initial 2–3 s. Overall, binocular rivalry was sensitive to both physical and imagery priming, with each influencing perceptual bias in early perception through distinct mechanisms.

To further examine whether priming influenced perceptual stability, we analysed the duration of the first dominant percept. In auditory streaming, the baseline bias toward integration precluded straightforward within-subject comparisons of congruent and incongruent durations. To address this, we calculated signed first-percept durations, assigning positive values to integrated and negative values to segregated percepts. Physical priming significantly reduced the average signed duration relative to baseline, with Bayesian analyses providing strong evidence for this effect. This finding suggests that segregated physical priming shifted early perceptual balance away from the integrated default. In contrast, imagery priming did not significantly alter signed first-percept duration, and Bayesian evidence supported the absence of an effect. Thus, although external auditory cues modulated both perceptual selection and early perceptual balance, internally generated auditory imagery did not measurably influence perceptual stability.

In binocular rivalry, no significant difference in duration was observed between prime-congruent and prime-incongruent first percepts in either physical or imagery priming conditions. Again, Bayesian results supported the null hypothesis, indicating that while priming influenced which percept emerged first, it did not affect how long that percept lasted. These findings suggest that priming, both sensory and imagery-based, primarily modulates initial perceptual selection, but does not extend to early perceptual stability, at least as indexed by first percept durations. This pattern further supports the idea that imagery may influence perceptual competition at the level of initial bias or perceptual access, rather than by altering the strength or persistence of perceptual representations.

Finally, we analysed whether individual differences in self-reported imagery vividness were associated with the strength of imagery priming. If vivid imagery reflects greater engagement of sensory representations, it should enhance the perceptual impact of internally generated content. Consistent with this prediction, we found a moderate positive correlation between PSIQ visual scores and the proportion of prime-congruent initial percepts in the binocular rivalry task. This suggests that participants with more vivid mental imagery were more susceptible to imagery-driven perceptual bias.

In contrast, no significant relationship was observed between auditory imagery vividness and perceptual outcomes in auditory streaming. Given the absence of imagery-based priming effects in the auditory paradigm, this null result is consistent with the broader pattern of findings. However, it remains unclear whether this reflects weaker coupling between auditory imagery and perception, or whether the intrinsic dynamics of auditory streaming constrain the detectability of imagery effects.

Collectively, the present findings refine our understanding of modality-specific imagery-perception interactions. First, physical priming influenced initial perceptual selection in both modalities, demonstrating that externally presented cues can bias early perceptual competition across sensory systems. In auditory streaming, this effect was detectable despite a bias towards initial integration, indicating that bottom-up cues can transiently overcome default perceptual tendencies. Second, imagery priming reliably biased perceptual selection only in the visual domain, suggesting that visual perceptual competition is more susceptible to internally generated representations than auditory streaming under the present parameters. Third, priming did not extend to altering percept duration in binocular rivalry and affected early perceptual balance only in auditory streaming via physical priming. Across paradigms, priming effects were therefore largely confined to perceptual onset.

One important consideration is the initial dominance of the integrated percept in auditory streaming. Although physical priming effects were detectable, the default toward integration possibly constrained the magnitude and temporal extent of modulation. Future studies could further reduce this baseline bias by employing stimuli closer to the temporal coherence boundary. Alternatively, one could extend the study of imagery priming to other auditory and visual bistable paradigms, because it has been shown that the neural mechanisms of perceptual bistability differ between auditory stream segregation and a bistable version of structure-from-motion paradigm. Thus it is possible that the relationship between auditory streaming and imagery is different from that between binocular rivalry and imagery.

Another limitation concerns the use of self-report measures to assess imagery vividness. While tools like the Plymouth Sensory Imagery Questionnaire (PSIQ)^48^; or the Vividness of Visual Imagery Questionnaire (VVIQ)^49–51^ are widely used, they are inherently subjective and susceptible to various biases, including social desirability, mood effects, and metacognitive distortions such as overconfidence or modesty^52–55^. Moreover, the overlap between memory and imagination may further complicate introspective ratings by blending prior experiences with constructed imagery^56,57^. The absence of an objective reference point for imagery vividness limits the interpretability of such self-reports^1,15^. These challenges underscore the need for developing more objective behavioural or neural indices of imagery vividness to complement self-reports and strengthen future research.

This study advances our understanding of how mental imagery interacts with perception across sensory modalities, a central question in theories of consciousness, predictive processing, and sensory simulation^1,2,4,58^. By using parallel paradigms in vision and audition, we demonstrate that while both physical and imagery cues can influence perception, their effects vary by modality, both in strength and temporal dynamics. The finding that imagery vividness predicts perceptual bias supports the idea that subjective imagery strength reflects functionally relevant top-down modulation, validating self-report tools and behavioural indices alike. Moreover, this work brings up the possibility that in different modalities, imagery may fit differently into the interplay between sensory systems and cognitive factors, such as attention and memory. These results therefore inform debates on the generative nature of perception and underscore the importance of studying imagery across modalities – not just in vision – to build a more comprehensive model of conscious experience.

In conclusion, our findings show that mental imagery can influence bistable perception in a modality-specific manner, with robust effects in vision but limited influence in audition – likely due to inherent differences in perceptual dynamics. The impact of imagery is strongest during perceptual onset and varies systematically with self-reported vividness, supporting its role as a meaningful and measurable contributor to conscious perception. These results highlight the value of modality-comparative approaches and provide behavioural evidence for the functional role of mental imagery in shaping perceptual experience.

### Methods

#### Participants

A total of *N* = 165 participants were recruited for the study: *N* = 72 participated in Auditory Streaming, *N* = 114 in Binocular Rivalry, with *N* = 21 appearing in both. Participants were selected using a convenience sampling method; the majority were university students who received course credit as an incentive for participation. Prior to the experiment, participants completed an informed consent form, a health declaration, and provided details about their optical prescription and demographic background, including age and gender.

Exclusion of participants was based on three distinct criteria, as detailed below. In Auditory Streaming, pure-tone audiometry was conducted in a sound-attenuated room using a calibrated screening audiometer (SA-7, Mediroll Kft., Debrecen, Hungary). For each test frequency, tones were initially presented at a clearly audible intensity level and subsequently decreased in a stepwise manner. The hearing threshold was defined as the lowest intensity (dB HL) at which the participant reliably detected the tone at a given frequency. Thresholds were determined separately for the right and left ears across all tested frequencies. Participants were excluded if the interaural threshold difference exceeded 15 dB at any frequency and/or if the hearing threshold exceeded 25 dB at any tested frequency in either ear. Based on these criteria, 10 participants were excluded.

Furthermore each condition concluded with a 30-second sequence – specifically, three 10-second segments (following 60 s of stimulus presentation) – that was unambiguously designed to be perceived as either segregated or integrated. This sequence served as a control to verify whether participants correctly remembered the baseline auditory sequences and were attentively reporting their perceptual experience. For a 10-second segment to be considered valid, participants had to indicate the correct percept (segregated or integrated) at least 75% of the time. For an entire condition to be accepted, at least two out of the three 10-second segments had to meet this 75% accuracy threshold. If this criterion was not met, only the specific trial was excluded.

In Binocular Rivalry, participants’ stereoscopic vision was assessed using the Super Stereoacuity Timed Tester (Wayne Engineering Co. & Vision Extension, 1991), resulting in the exclusion of 4 participants due to inadequate stereoacuity, as indicated by failing the first four levels of the test. 2 additional participants were excluded from the Binocular Rivalry due to excessive movement, which prevented proper calibration. In total, 6 participants were excluded based on criteria specific to this paradigm.

After applying all exclusion criteria, the final sample consisted of *N* = 152 participants: *N* = 62 in Auditory Streaming, *N* = 108 in Binocular Rivalry, and *N* = 18 who participated in both. In Auditory Streaming, the mean age of the participants was M = 24.31 (SD = 3.97), and 78% of the sample identified as female, while in Binocular Rivalry the mean age of the participants was M = 22.16 (SD = 4.3), and 80.7% of the sample identified as female.

Based on the a priori power analysis, the sample size remained adequate for all tests even after the exclusions, with the exception of Binocular Rivalry, where the ANOVA would have required a larger number of participants per group. Detailed results are presented in the Supplementary Materials.

Ethical approval for this study was provided by the Ethical Committee of Pázmány Péter Catholic University (PPCU), Budapest, Hungary. All experiments were performed in accordance with relevant guidelines and regulations.

### Measurement of imagery vividness

To assess the vividness of imagery across different sensory modalities, we used the Hungarian version of the Plymouth Sensory Imagery Questionnaire (PSIQ; Andrade et al., 2014^48^). The questionnaire consists of 35 items, divided into seven subscales (5 items per subscale), each targeting a specific modality: visual, auditory (sound), olfactory (smell), gustatory (taste), tactile (touch), bodily sensation, and emotional feeling. Participants respond to each item using an 11-point Likert scale ranging from 0 (“no image at all”) to 10 (“image as clear and vivid as real life”), depending on how vividly they can imagine the given sensory experience.

Separately, in Binocular Rivalry and in Auditory Streaming, participants were divided into three groups based on the quartiles of their total scores on the corresponding subscale: the lowest (Q1), the intermediate (Q2 + Q3), and the highest (Q4) imagery vividness group.

### Design of the experiment, paradigms employed, and stimulus parameters

#### Auditory streaming

Auditory stimuli were presented binaurally using Sennheiser HD-600 headphones connected to a Mackie 802 VLZ4 mixer driven by an ESI MAYA22 USB sound card, connected to a PC. Stimulus delivery was controlled via custom scripts implemented in MATLAB (version 8.3, 2014a)^59^ and Psychtoolbox-3 (version 3.0.14)^60^.

The ambiguous auditory stimulus consisted of two sinusoidal pure tones: tone A at 400 Hz and tone B at 599.3 Hz. The tones were arranged in a repeating galloping ABA_ pattern. Each tone had a duration of 75 ms and was followed by a 150 ms silent interval. Stimuli were presented at an average sound pressure level of 60 dB SPL.

In addition to the ambiguous sequence, several unambiguous sequences were constructed. The integrated sequence preserved the same temporal structure but reduced the frequency separation, with tone A at 400 Hz and tone B at 434 Hz, strongly promoting perceptual integration. The extremely segregated sequence maintained the temporal structure but increased frequency separation to 400 Hz and 900 Hz, strongly promoting segregation. Two additional segregated control sequences were created by attenuating either the A tone or the B tone of the ambiguous stimulus while preserving the original temporal and frequency structure. These attenuated sequences were used during control segments to verify task compliance and perceptual discrimination.

Prior to training, participants completed a learning phase to familiarize themselves with the perceptual categories. They were presented with one-minute examples of the extremely integrated sequence, the extremely segregated sequence, and the two attenuated segregated sequences. All learning sequences followed the same temporal structure as the ambiguous stimulus. Any of these sequences could be repeated upon request to ensure perceptual clarity.

Participants’ perceptual experience was categorized as either integrated or segregated auditory streaming. An integrated percept corresponded to the experience of a single coherent stream incorporating both frequencies, whereas a segregated percept reflected the experience of two distinct parallel streams, each with a single frequency. Participants were instructed to continuously report their perceptual state by selecting between the two alternatives – integrated or segregated by pressing the left or right mouse button, respectively; if they were unable to make a decision, they were instructed to release both buttons. The instructions emphasized that there were no correct or incorrect responses; rather, the focus of the study was solely on the participants’ subjective perceptual experience.

Each training trial included 60 s of ambiguous stimulation followed by three 10-second unambiguous control segments, comprising one integrated and two attenuated segregated sequences presented in randomized order. Participants continuously reported their perceptual state. Performance during the control segments was evaluated, and if accuracy fell below 80%, the training trial was repeated upon request or until satisfactory performance was achieved.

Each experimental trial consisted of a 60-second ambiguous sequence followed by three 10-second unambiguous control segments presented in randomized order. The control segments always included one integrated and two attenuated segregated sequences. Based on this trial structure, four experimental conditions were implemented.

In the baseline condition, participants were presented with the standard 60-second ambiguous sequence followed by the three control segments without any preceding manipulation.

This was followed by the pre-cueing conditions: the segregated priming sequence was derived from the ambiguous stimulus by silencing the higher tones, resulting in a temporally identical sequence containing only repeated lower tones.

In the segregated physical priming condition, each trial began with a 10-second segregated priming sequence, followed by a 1-second silent interval. This was immediately followed by the 60-second ambiguous sequence and the three control segments.

Two additional conditions employed a shorter segregated priming manipulation followed by a longer silent interval. In both cases, each trial began with a 5-second segregated priming sequence, followed by a 10-second silent interval before the onset of the ambiguous stimulus and subsequent control segments. Based on Beauvois and Meddis (1997)^61^, we expected that any potential effect of the previously presented physical priming would dissipate after a 10-second delay. Accordingly, in these conditions the earlier unambiguously segregated stimulus was not expected to influence the initial perception of the subsequent ambiguous stimulus. The two conditions differed only in the instructions given for the silent interval. In the physical priming condition with extended delay, participants were instructed to remain passive during the 10-second silence. In the imagery priming condition, participants were instructed to imagine as vividly as possible the tone pattern they had just heard during the 5-second priming phase, maintaining the mental representation throughout the silent interval.

Data acquisition was organized into two experimental blocks separated by a break during which participants could leave the testing room. In the first block, participants completed three trials of the baseline condition followed by five trials of the 10-second segregated physical priming condition. After the break, participants completed three additional baseline trials, followed by five trials of the physical priming condition with extended delay and five trials of the imagery priming condition. All other procedural aspects remained identical to those described above.

### Binocular rivalry

The experimental setup followed the design developed by our group earlier^15,38,39^. In Binocular Rivalry stimuli were displayed on LCD monitors (refresh rate: 120 Hz) using a dichoptic setup (spatial frequency: 0.26 cycles/degree, resolution: 48 pixels/°). Gaze data was collected using the EyeLink 1000 Plus system through a cold mirror stereoscope, ensuring accurate binocular tracking^62^. Participants sat with head support to minimize movement. Each grating was rectangular, framed with a random texture to aid binocular fusion, and subtended 15.2° (width) × 8.4° (height) of visual angle. The gratings had 50% contrast, 8.7 Hz temporal frequency, and moved at 33.5°/s (1600 pixels/s). Stimuli were generated in MATLAB R2019b^59^ using the Psychophysics Toolbox^63^.

After testing stereoscopic vision, participants began with a practice trial to familiarize themselves with the binocular rivalry task. During this phase (and the further experimental phases), they were instructed to pay attention to the gratings they saw and let their eyes follow it. As perceptual dominance was objectively measured via optokinetic nystagmus, no behavioral responses or verbal reports were required.

In the experiment setup there were three blocks: a physical priming, an imagery priming, and a conceptual priming block, although the latter was not analysed in this study (see Welker et al., 2025^15^). The two observed priming blocks – physical and imagery – a visual imagery priming in which participants were asked to visualize the gratings, and a physical priming in which a clear directed stimulus was seen on the displays. Each block began with a 25-second baseline measurement, followed by 12 priming trials with semi-randomized motion directions. While the overall proportion of directions was fixed, the sequence order varied randomly to avoid predictability.

Each priming trial followed a consistent structure: started with a 2-second stimulus presenting the prime direction, followed by a 5-second phase. In the visual imagery priming condition, participants were instructed to continuously visualize the previously seen primed direction as a picture or a video. In the physical priming condition, a further 5-second identical stimulus to the previously presented cue was shown. Each block ended with a 10-second binocular rivalry phase, which was used as the basis for further analysis. Based on prior findings^15^, the priming effect typically lasts around 1.5 s; therefore, the 5-second priming duration was sufficient to ensure that any influence on rivalry perception stemmed from the priming condition itself rather than from residual effects of the cue stimulus.

### Data analysis – binocular rivalry

Optokinetic nystagmus is a reflexive eye movement triggered by motion, where the direction of smooth pursuit reflects perceived motion – even under conditions of competing cues. As in our previous study^15^, horizontal eye movements were recorded at a 1000 Hz sampling rate, and the perceived direction over time was analyzed using the Cumulative Smooth Pursuit method^39^. This method first removes artifacts (e.g., those caused by blinks) and then identifies slow pursuit segments based on criteria such as low velocity, low acceleration, and a minimum duration. After detecting and aligning these segments, gaps are filled through interpolation, and the dataset is resampled using a ‘bagging’ technique. The result is an estimated eye velocity profile for each time point, represented with median values and corresponding confidence intervals. Dominance periods are defined as intervals during which the 95% confidence interval consistently stays above or below a predetermined gaze velocity threshold, while all other intervals are classified as either perceptual transitions or mixed percepts^39^.

## Supplementary Information

Below is the link to the electronic supplementary material.

Supplementary Material 1

## Data Availability

The datasets used in this study and Supplementary Material are available at the Open Science Framework (OSF) platform at the following address: [ https://osf.io/eg4ax/overview?view_only=1d2bdbf79f2247e8903b0a053c81af33).
