# Supplementary material for: Mental imagery modulates bistable perception in a modality-specific manner
Source: Sci Rep. 2026 Mar 19;16:14230. doi: 10.1038/s41598-026-44578-2 (PMC13139475; doi:10.1038/s41598-026-44578-2)
Supplement: Supplementary file 1 — Supplementary Material 1 [file 41598_2026_44578_MOESM1_ESM.pdf]

Supplementary Materials for

Mental Imagery Modulates Bistable Perception in a Modality-Specific Manner

Luca Verebélyi [1], Ágnes Welker [1,2], Kökény Kovács-Deák [1], Ferenc Gombos [3,4], Gábor Péter Háden [1], István Winkler [1] & Ilona Kovács [4,5]

[1] HUN-REN Research Centre for Natural Sciences, Budapest, Hungary

[2] Semmelweis University Doctoral School, Budapest, Hungary

[3] Pázmány Péter Catholic University, Budapest, Hungary

[4] HUN-REN-ELTE-PPKE Adolescent Development Research Group, Budapest, Hungary

[5] Faculty of Education and Psychology, Eötvös Loránd University, Budapest, Hungary

- The symbols , **, and *** indicate statistical significance levels, where p < 0.05, p < 0.01, and p < 0.001.*
- All statistical analyses were conducted using JASP software[1], except for the power analyses, which were conducted with G*Power[2].

Table 1

Descriptive Statistics for signed length of first perception [ms] Across Conditions in the Auditory Streaming

	Baseline	Physical Prime	Physical Prime with extended delay	Imagery Prime
Valid	61	61	60	60
Missing	1	1	2	2
Median	6517	712	2463	4144
Mean	3239	-2723	1809	2017
Std. Error of Mean	1565	1774	1206	1386
Std. Deviation	12221	13860	9341	10740
Shapiro-Wilk	0.944	0.942	0.957	0.953
P-value of Shapiro-Wilk	0.008	0.006	0.032	0.023
Minimum	-34380	-38200	-29090	-29410
Maximum	29590	18980	16420	28280

Note. All conditions significantly deviated from a normal distribution (Shapiro–Wilk test, p < .05), which justifies using non-parametric analyses in subsequent statistical tests.

Table 2
Descriptive Statistics for First Percepts' Duration Across Conditions in the Binocular Rivalry

	Imagery – Prime-Congruent Percepts	Imagery – Prime-Incongruent Percepts	Physical – Prime-Congruent Percepts	Physical – Prime-Incongruent Percepts
Valid	106	105	102	107
Missing	3	4	7	2
Median	1295.917	1148.667	1102.917	1129.750
Mean	1419.091	1395.435	1369.414	1330.898
Std. Error of Mean	72.567	88.260	114.236	63.279
Std. Deviation	747.122	904.394	1153.724	654.562
Shapiro-Wilk	0.852	0.788	0.639	0.895
P-value of Shapiro-Wilk	< .001***	< .001***	< .001***	< .001***
Minimum	225.000	365.333	28.000	270.000
Maximum	5687.625	5664.000	9998.000	3840.000

Note. All conditions significantly deviated from normality (according to Shapiro-Wilk Test, $p < 0.001$), justifying the use of non-parametric tests in subsequent analyses.

Table 3

Wilcoxon Signed-Rank Test Results signed length of First Percept [ms] Across Conditions in the Auditory Streaming

			W	z	p	Rank-Biserial Correlation	SE Rank-Biserial Correlation	BF_{10}	$\hat{R}$
Baseline	-	Physical Prime	1380	3.121	0.002	0.460	0.146	29.564	1.038
Physical Prime with extended delay	-	Imagery Prime	905	-0.074	0.944	-0.011	0.147	0.143	1.001

Note. As Table 1 shows a significant deviation from normality, Wilcoxon Signed-Rank Test was applied, revealing to insignificant results in both conditions (in case of the Physical Condition, W = 1380, Z = 3.121, p = .002, rank-biserial correlation = 0.460 (SE = 0.146), while in Imagery Condition W = 905, Z = -0.074, p = .944, rank-biserial correlation = -0.011 (SE = 0.147). The Bayesian Wilcoxon signed-rank test showed strong evidence supporting the hypothesis in physical priming (BF_{10} = 29.564) and moderate evidence supporting the null hypothesis in the imagery priming (BF_{10} = 0.143). The Gelman–Rubin convergence values ($\hat{R} \approx 1.000$) suggest that the MCMC chains converged properly, so the results are reliable.

Table 4

Wilcoxon Signed-Rank Test for First Perception Duration Across Conditions in the Binocular Rivalry

Measure 1		Measure 2	W	z	p	Rank-Biserial Correlation	SE Rank-Biserial Correlation	BF_{10}	$\hat{R}$
Imagery – Prime-Congruent Percepts	-	Imagery – Prime-Incongruent Percepts	2966.000	0.947	0.344	0.108	0.113	0.126	1.001
Physical – Prime-Congruent Percepts	-	Physical – Prime-Incongruent Percepts	2311.500	-0.894	0.372	-0.103	0.114	0.123	1.003

Note. In this analyses for each participant their prime-directed and non-prime directed first percepts were averaged. As Wilcoxon Signed-Rank Test shows, no significant result was found (in the imagery condition, W = 2966.000, Z = 0.947, p = .344, rank-biserial correlation = 0.108 (SE = 0.113), while in physical condition W = 2311.500, Z = -0.894, p = .372, rank-biserial correlation = -0.103 (SE = 0.114).

Table 5a

Contingency table for First Percepts

Auditory Streaming					Binocular Rivalry				
		First Perception Type					First Perception Type		
Condition		Integrated	Segregated	Total	Condition		Right	Left	Total
Baseline	Count	280	100	380	Left Physical Priming	Count	374	114	488
	%	74 %	26%	100 %		%	77 %	23 %	100 %
Physical Priming	Count	168	128	296	Right Physical Priming	Count	315	412	727
	%	57%	43%	100 %		%	43 %	57 %	100 %
Physical Priming with extended delay	Count	194	114	308	Left Imagery Priming	Count	222	264	486
	%	63%	37 %	100 %		%	46%	54 %	100 %
Imagery Priming	Count	193	107	300	Right Imagery Priming	Count	433	308	741
	%	6494 %	6 36%	100 %		%	58 %	42 %	100%

Table 5b

Chi-square analysis of first percepts

	Auditory Streaming					Binocular Rivalry				
Condition	Value	df	p	Cramer's V	BF_{10}	Value	df	p	Cramer's V	BF_{10}
Physical Priming X^2	***21.33***	***1***	***<.001***	***0.178***	***3737***	**137.088**	**1**	**< .001**	**0.335**	**$3.492*10^{28}$**
Imagery Priming X^2	*0.119*	*1*	*0.730*	*0.014*	*0.103*	**19.189**	**1**	**< .001**	**0.125**	**1062.612**

Note. As Table 5 shows, only the physical primingin the Auditory Streaming significant ($\chi^2(1, N = 676) = 21.33$, $p < .001$, Cramer's V = 0.178, $BF_{10} = 3737$) while in imagery prime there was no significant difference ($\chi^2(1, N = 608) = 0.119$, $p = .730$, Cramer's V = 0.014, $BF_{10} = 0.103$) On the other hand, Binocular Rivalry led to significant results in both cases (in physical prime: $\chi^2(1, N = 1215) = 137.088$, $p > .001$, Cramer's V = 0.335, $BF_{10} = 3.492*10^{28}$; while in imagery prime $\chi^2(1, N = 1227) = 19.189$, $p > .001$, Cramer's V = 0.125, $BF_{10} = 1062.612$) justifying subsequent statistical tests.

Table 6

Spearman's Correlation and Bayesian Kendall's Tau Between the Auditive Subscale of the PSIQ and the ratio of Prime-Congruent First Percepts in the Imagery Condition of the Auditory Streaming

			Spearman's rho	p	Effect size (Fisher's z)	SE Effect size	Kendall's tau B	BF_{10}
Auditive Subscale of the PSIQ	-	Imagery priming– Ratio of Prime-Congruent First Percepts	-0.060	0.647	-0.060	0.132	-0.053	0.200

Note. No significant association was found between participants' Auditive Imagery ability (PSIQ – Auditive Subscale) and the proportion of prime-congruent first percepts in the imagery priming condition.

Table 7

Spearman's Correlation and Bayesian Kendall's Tau Between the Visual Subscale of the PSIQ and the ratio of Prime-Congruent First Percepts in the Imagery Condition of the Binocular Rivalry

			Spearman's rho	p	Effect size (Fisher's z)	SE Effect size	Kendall's tau B	BF_{10}
Visual Subscale of the PSIQ	-	Imagery priming– Ratio of Prime-Congruent First Percepts	0.327	< .001***	0.339	0.100	0.233	68.312

Note. A significant positive association was found between participants' Visual Imagery ability (PSIQ – Visual Subscale) and the proportion of prime-congruent first percepts in the imagery priming condition, Spearman's $\rho = .327$, $p < .001$, Fisher's *$z = 0.339$, $SE = 0.100$. Bayesian Kendall's tau showed a positive correlation between visual subscale of the PSIQ and prime-congruent perception in the imagery condition of Binocular Rivalry (Kendall's tau B = 0.233, $BF_{10} = 68.31$), indicating strong evidence for the alternative hypothesis.*

In the following analyses, we examine the data along quartiles based on the auditive subscale of the PSIQ. q1 indicates low auditive imagery ability, while q4 refers to hyperphantasia (see more details in the results).

Table 8.

Kruskal-Wallis Test for proportion of prime-congruent first percept in imagery priming in Auditory Streams between imagery groups (q1, q2+q3,q4)

Factor	Statistic	df	p
**Imagery groups**	**1.303**	**2**	**.521**

Note: Kruskal-Wallis finds no significant difference between auditory imagery groups and imagery priming in auditory streams.

In the following analyses, we examine the data along quartiles based on the visual subscale of the PSIQ. Q1 indicates low visual imagery ability, while Q4 refers to hyperphantasia (see more details in the results).

Table 9.A.

Test for Equality of Variances (Levene's) for Prime-Congruent Percepts in Imagery Condition of Binocular Rivalry

F	df1	df2	p
3.883	3.000	104.000	0.011**

Participants were grouped based on their PSIQ vision subscale scores into four quartile-based groups. Levene's test showed significant variance differences (F(3, 104) = 3.883, p = 0.011), so Welch's correction was applied in the ANOVA.

Table 9.B.

ANOVA for Prime-Congruent Percepts in Imagery Condition of Binocular Rivalry

Homogeneity Correction	Cases	Sum of Squares	df	Mean Square	F	p	ω²
Welch	quartile	0.595	3.000	0.198	9.081	< .001***	0.108
	Residuals	3.837	56.650	0.068			

Note. ANOVA with Welch homogeneity correction suggest significant group differences (F(3, 56.650) = 9.081, p < .001, with a partial omega squared (ω²) of 0.108), suggesting a moderate effect size.

Table 9.C.

Post Hoc Comparisons for Prime-Congruent Percepts in Imagery Condition of Binocular Rivalry

		Mean Difference	SE	df	t	Cohen's d	p_{tukey}
q1	q2	-0.183	0.054	104	-3.400	-0.954	0.005 **
	q3	-0.097	0.051	104	-1.887	-0.505	0.240
	q4	-0.180	0.052	104	-3.469	-0.936	0.004 **
q2	q3	0.086	0.053	104	1.628	0.449	0.368
	q4	0.003	0.053	104	0.065	0.018	1.000
q3	q4	-0.083	0.051	104	-1.627	-0.431	0.368

Note. As Table 9.C. shows, the only significant differences between the groups were suggested between q1 and q2 (the mean difference was -0.183 (SE = 0.054), t(104) = -3.400, Cohen's d = -0.954, p = 0.005), as well as between q1 and q4 (the mean difference was -0.180 (SE = 0.052), t(104) = -3.469, Cohen's d = -0.936, p = 0.004). (P-value adjusted for comparing a family of 4 estimates.)

Table 10.A.

Bayesian Model Comparison Across PSIQ Visual Imagery Quartiles

Models	P(M)	P(M|data)	BF_M	BF_{10}	error %
quartiles	0.500	0.950	18.812	1.000	
Null model	0.500	0.050	0.053	0.053	0.005

Note. As Table 10 shows, Bayesian model comparison supported a strong evidence for the main model over the null model (BF_{10} = 18.81, P = .95, BF_{10} = 18.81, error = 0.5%).

Table 10.B.

Post Hoc Comparisons Across PSIQ Visual Imagery Quartiles

		Prior Odds	Posterior Odds	$BF_{10, U}$	error %
q1	q2	0.414	346.595	836.755	1.424×10^{-5}
	q3	0.414	0.430	1.038	0.009
	q4	0.414	35.509	85.726	3.730×10^{-8}
q2	q3	0.414	0.283	0.683	0.008
	q4	0.414	0.116	0.279	0.008
q3	q4	0.414	0.240	0.580	0.009

Note.This table presents post hoc Bayesian t-tests comparing PSIQ visual imagery quartiles. Posterior odds have been adjusted for multiple comparisons by setting the prior probability of the null hypothesis to 0.5 across all tests (Westfall, Johnson, & Utts, 1997). A default Cauchy prior (location = 0, scale = 1/√2) was used. BF_{10} values indicate evidence in favor of the alternative hypothesis; "U" denotes uncorrected Bayes Factors. Error percentages reflect the numerical accuracy of the estimation.

Table 11.A.
Test for Equality of Variances (Levene's) for PSIQ Visual Imagery Quartiles (with Q2 and Q3 Combined)

F	df1	df2	p
3.022	2.000	105.000	0.053

Note. Levene's test for homogeneity of variances was not significant, F(2, 105) = 3.02, p = .053, indicating that no correction was needed for the ANOVA.

Table 11.B.
ANOVA Across PSIQ Visual Imagery Quartiles (with Q2 and Q3 Combined)

Cases	Sum of Squares	df	Mean Square	F	p	ω²
Q1 – (Q2 + Q3) – Q4	0.497	2	0.249	6.637	0.002**	0.095
Residuals	3.935	105	0.037			

Type III Sum of Squares was used. The grouping was based on PSIQ visual imagery quartiles, with Q2 and Q3 combined. The result shows a significant effect across groups, F(2, 105) = 6.64, p = .002, with a medium effect size (ω² = 0.095).

Table 11.C.
Post Hoc Comparisons Across PSIQ Visual Imagery Quartiles (with Q2 and Q3 Combined)

		Mean Difference	SE	df	t	Cohen's d	p_{tukey}	$p_{scheffe}$
q1	q2 + q3	-0.136	0.046	105	-2.971	-0.702	0.010*	0.014*
	q4	-0.180	0.052	105	-3.442	-0.928	0.002**	0.004**
q2 + q3	q4	-0.044	0.045	105	-0.967	-0.226	0.599	0.628

Note. Tukey's HSD and Scheffé tests revealed significant differences between Q1 and Q2+Q3 (t(105) = -2.97, p = .010/.014, d = -0.70) and between Q1 and Q4 (t(105) = -3.44, p = .002/.004, d = -0.93). No significant difference was found between Q2+Q3 and Q4 (t(105) = -0.97, p = .599/.628, d = -0.23).

Table 12

Bayesian Model Comparison Based on PSIQ Visual Imagery Quartiles (Q2 and Q3 Combined)

Models	P(M)	P(M|data)	BF_M	BF_{10}	error %
kvart3	0.500	0.940	15.719	1.000	
Null model	0.500	0.060	0.064	0.064	0.013

Note. Bayesian model comparison showed strong evidence in favor of the model based on PSIQ visual imagery quartiles with Q2 and Q3 combined (BF_{10} = 15.72, P(M|data) = .94), compared to the null model (BF_{10} = 0.064, P(M|data) = .06). Estimation error was 1.3%.

Table 13

Bayesian Model Comparison Across PSIQ Visual Imagery Quartiles (Q2 and Q3 Combined)

		Prior Odds	Posterior Odds	$BF_{10, U}$	error %
q1	q2 + q3	0.587	6.768	11.522	3.027×10^{-7}
	q4	0.587	50.355	85.726	3.730×10^{-8}
q2 + q3	q4	0.587	0.199	0.339	0.014

Note. The posterior odds have been corrected for multiple testing by fixing to 0.5 the prior probability that the null hypothesis holds across all comparisons (Westfall, Johnson, & Utts, 1997). Individual comparisons are based on the default t-test with a Cauchy (0, r = 1/sqrt(2)) prior. The "U" in the Bayes factor denotes that it is uncorrected.

All of the following power analyses were tested using the G*Power.

Figure 1

Power analysis for χ^2 tests – Goodness-of-fit (contingency tables)

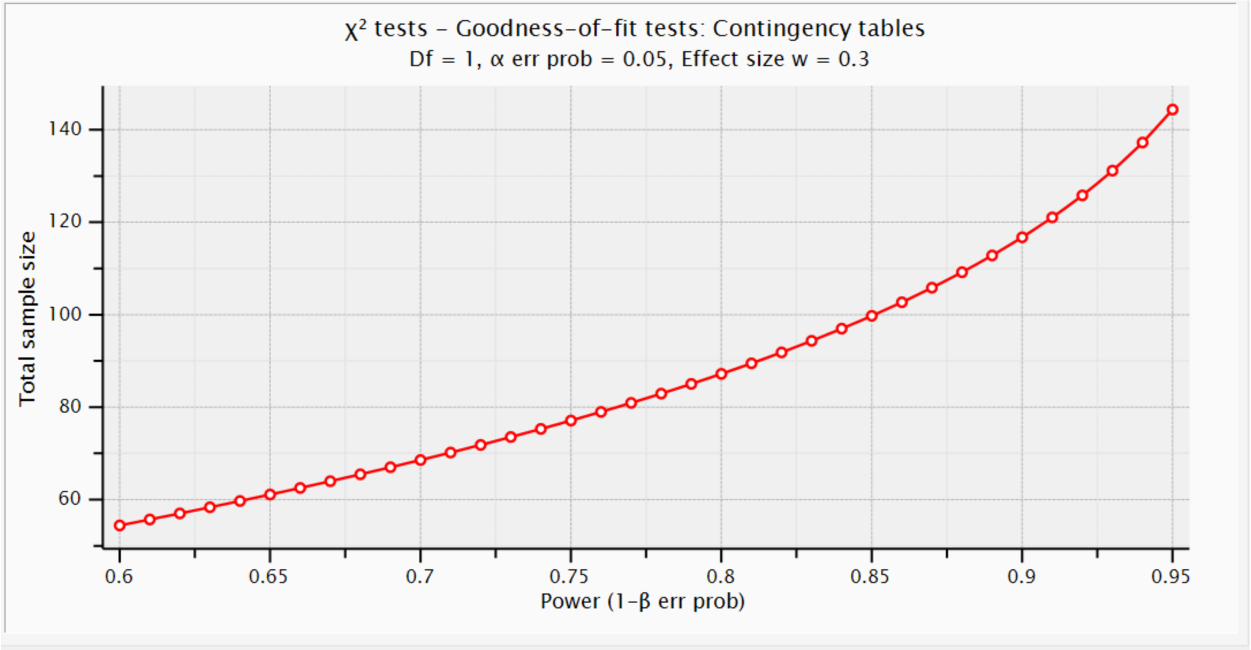

Note. *The graph shows the relationship between statistical power (1 - β error probability) and required total sample size for a χ^2 goodness-of-fit test with 1 degree of freedom, $\alpha = 0.05$, and effect size w = 0.3. As statistical power increases, the total sample size required increases exponentially, reaching approximately 145 participants at 95% power.*

Figure 2

Power analysis for t tests – Wilcoxon signed-rank test (matched pairs)

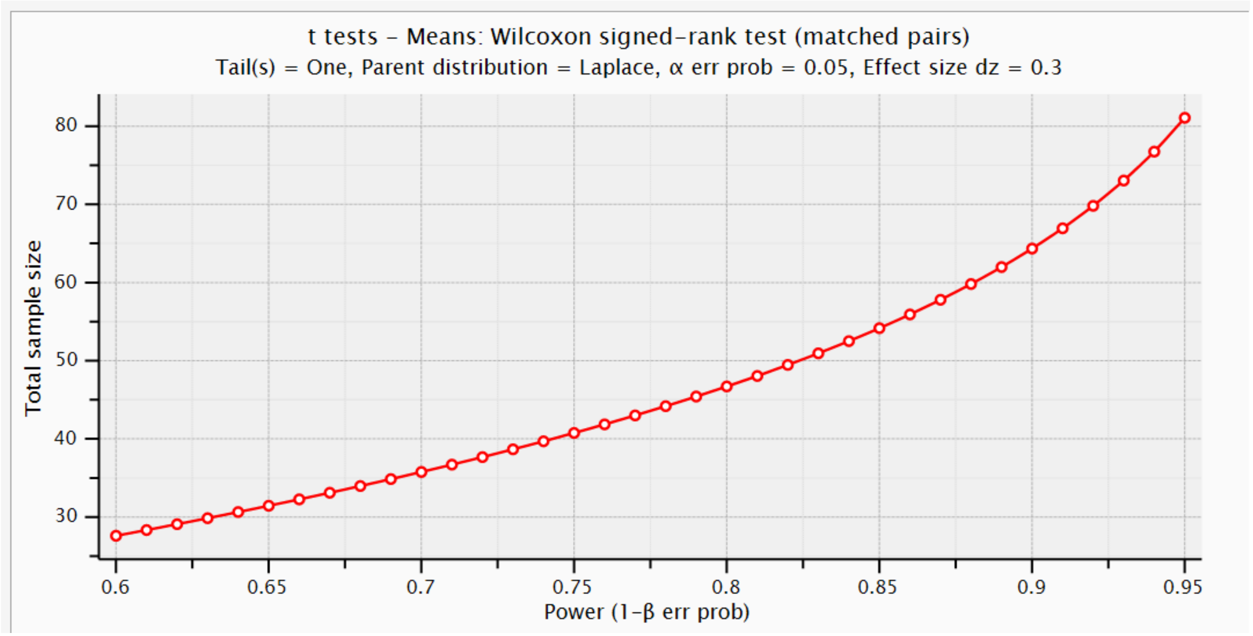

Note. *The graph displays the required total sample size for the Wilcoxon signed-rank test under a one-tailed hypothesis, Laplace parent distribution, α = 0.05, and effect size dz = 0.3. The sample size requirement increases with desired power, but remains notably lower than that of the χ^2 test, reaching about 82 participants at 95% power.*

Figure 3

Power analysis for t tests – Correlation (point-biserial model).

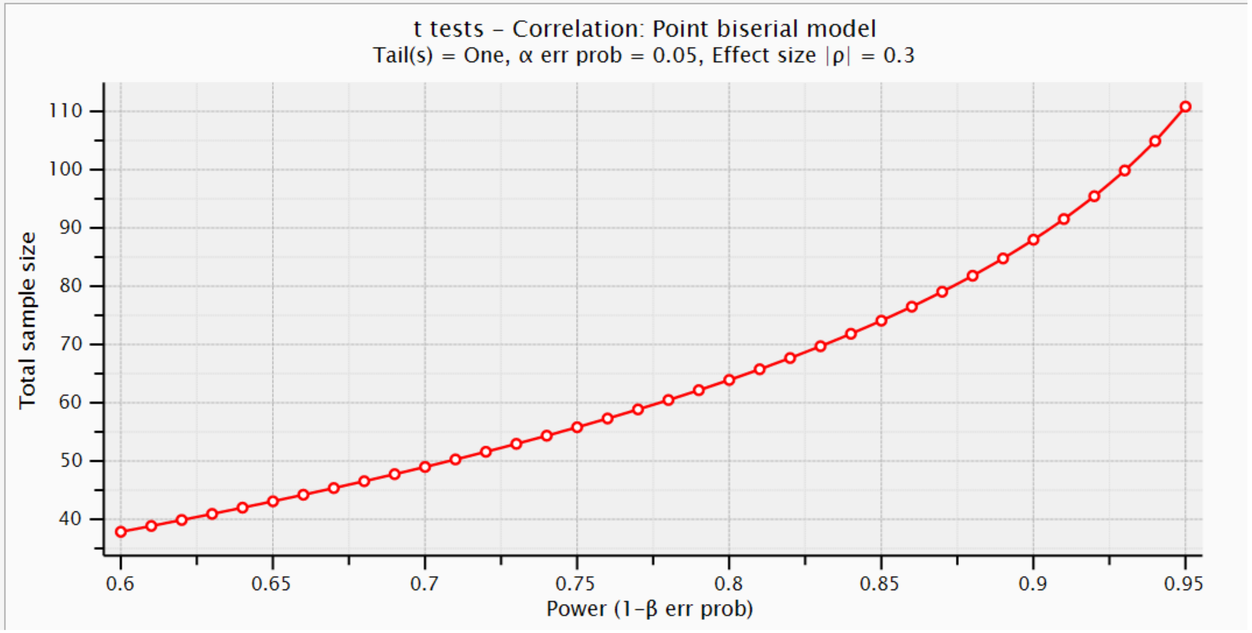

Note. The graph shows the required total sample size as a function of statistical power (1 - β) for a one-tailed t-test based on a point-biserial correlation. Parameters: α = 0.05, effect size |ρ| = 0.3. The sample size increases with desired power, ranging from approximately 60 participants (power = 0.7) to around 110 (power = 0.95).

Figure 4

Power analysis for F tests – ANOVA (fixed effects, omnibus, one-way).

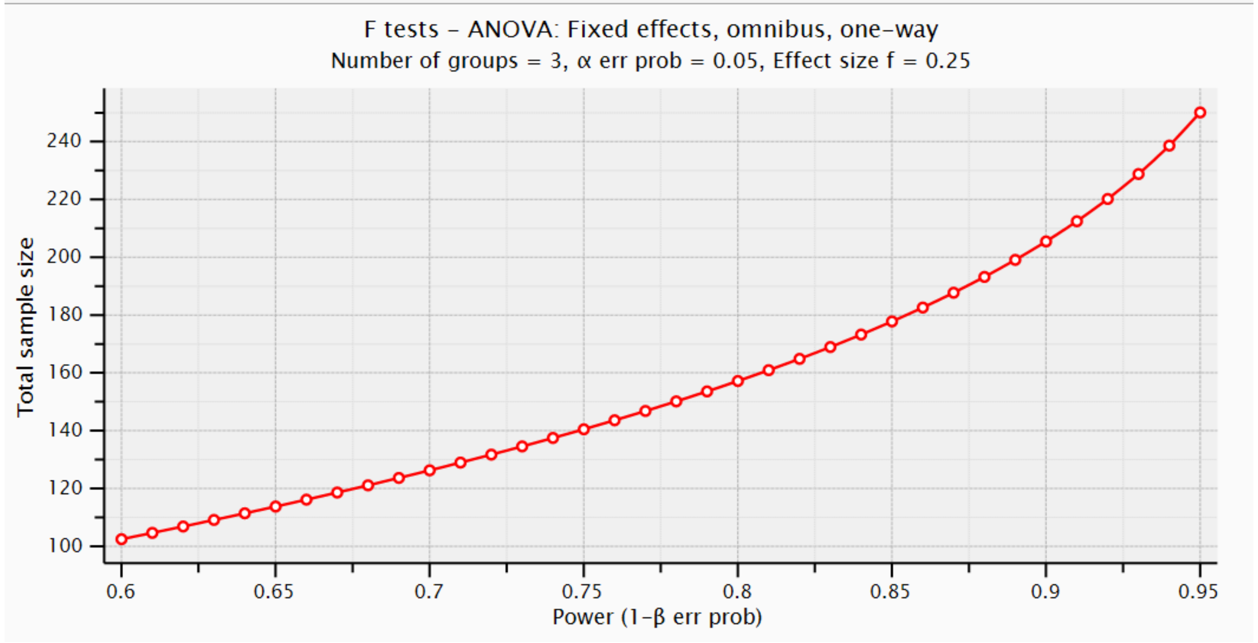

Note. The graph displays the relationship between statistical power and total sample size for a one-way ANOVA with 3 groups. Parameters: α = 0.05, effect size f = 0.25. The required sample size rises with increasing power, from about 145 participants (power = 0.7) to roughly 245 (power = 0.95).

Figure 5

Power analysis for F tests – ANOVA (fixed effects, omnibus, one-way) with four groups.

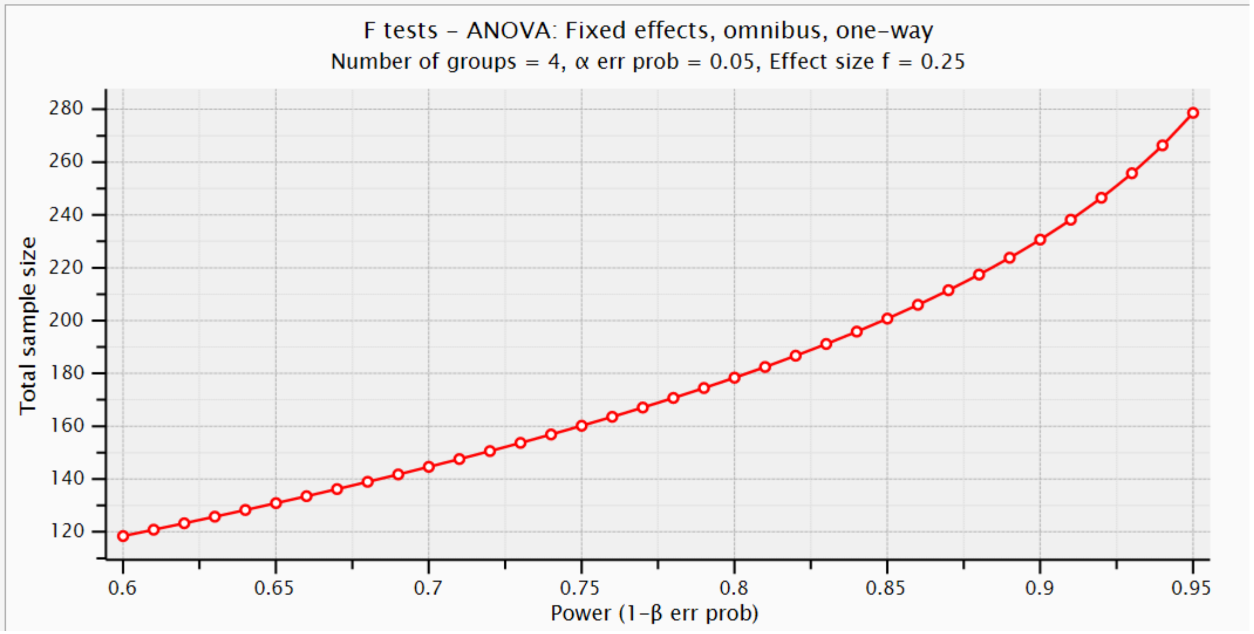

Note. The graph shows the required total sample size as a function of statistical power (1 - β) for a one-way fixed effects ANOVA with 4 groups, α = 0.05, and effect size f = 0.25. As desired power increases, the sample size rises from approximately 125 participants (power = 0.6) to about 275 participants (power = 0.95).

References

[1]JASP Team. (2024). *JASP* (Version 0.19.0) [Computer software]. https://jasp-stats.org/

[2] Faul, F., Erdfelder, E., Buchner, A., & Lang, A.-G. (n.d.). *G*Power (Version 3.1) [Computer software]. Heinrich Heine University Düsseldorf. https://www.psychologie.hhu.de/arbeitsgruppen/allgemeine-psychologie-und-arbeitspsychologie/gpower

Figure 6

In the analysis of the PSIQ scores, radar charts were constructed to visualize four groups based on quartiles of the total PSIQ score (a), the visual subscale score (b), and the auditory subscale score (c). From panel (a), where groups are based on the total score, the quartiles appear to be clearly distinguishable in terms of individual perceptual experiences (represented as axes of the radar chart). This suggests that the different sensory modalities may co-vary to some extent, and that the PSIQ can serve as a moderately effective tool for assessing the vividness of mental imagery across modalities. However, as shown in panels (b) and (c), where grouping is based on the visual and auditory subscales respectively, the separation between quartiles becomes less distinct. The overlap observed between groups indicates that when subgrouping is based on a single modality, such as vision or audition, differentiation between participants is less reliable. This implies that despite targeting similar constructs, individual subscale scores do not strongly predict performance in other modalities.

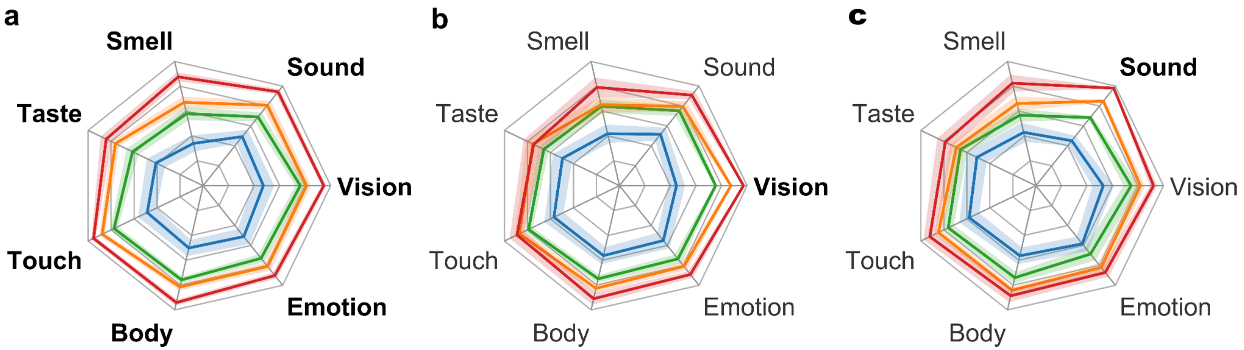

Figure 6. Spider plot showing the total scores across the 7 subscales of the PSIQ. **a.** Participants were grouped into four quartile-based groups according to their total PSIQ score. **b.** Participants were grouped into four quartile-based groups according to their visual subscale score. **c.** Participants were grouped into four quartile-based groups according to their auditory subscale score. Q1 (quartile group with the lowest score) – blue, Q2 – green, Q3 – orange, Q4 (quartile group with the highest score) – red. Shaded area: 95% confidence interval.

The present study underwent substantial conceptual and methodological refinement during the revision process. The Auditory Streams paradigm retained its original conceptual foundation; however, its experimental implementation and analytical framework were extensively revised. Data collection was repeated.
A detailed description of the revised implementation of the Auditory Streams paradigm is provided in the present Supplementary Methods to preserve the narrative flow of the main manuscript while ensuring full transparency and reproducibility.

“Design of the experiment, paradigms employed, and stimulus parameters

Auditory Streams

In Auditory Streams, auditory stimuli were presented binaurally using Superlux HD-681 headphones, driven by an ESI MAYA22 USB sound card, connected to an HP ProBook 450 G3 laptop. Stimulus delivery was controlled via custom scripts implemented in MATLAB[61].

Sinusoidal tones were arranged in an alternating sequence of two tones (A and B), where “A” and “B” denote two different frequencies (“A” tone 400 Hz and “B” tone 504 Hz). Each tone lasted 75 ms, including 10 ms linear onset and offset ramps to avoid abrupt sound transitions. The tones were presented at an average sound pressure level of 45 dB SPL.

Participants’ perceptual experience was classified into two distinct categories: integrated and segregated auditory streams. An integrated percept corresponds to the experience of a single coherent stream combining both low and high tones, whereas a segregated percept reflects the simultaneous but separate perception of low and high tones as distinct auditory streams.

Participants were instructed to continuously report their perceptual state by selecting between the two alternatives – integrated or segregated by pressing the left or right mouse button, respectively; if they were unable to make a decision, they were instructed to release both buttons. The instructions emphasized that there were no correct or incorrect responses; rather, the focus of the study was solely on the participants’ subjective perceptual experience.

In Auditory Streams, the experiment began with a demonstration and practice phase, followed by five experimental conditions. Each trial consisted of a 120-second ambiguous sequence followed by a 30-second control segment. The latter served to verify participants’ understanding of the task and their memory of the baseline auditory sequences. During the demonstration phase, participants were first introduced to the ambiguous auditory streams sequence. Subsequently, in the practice session, they were required to continuously report their perception of the ambiguous sequence as either integrated or segregated. We analyzed the proportion of correct tone choices made by participants during the control segment at the end of the practice session. The tones were either clearly integrated or segregated, based on the principle described above, and we monitored the proportion of correct responses. If a participant’s accuracy fell below 80% during the control phase, they were required to repeat the practice until their performance exceeded this criterion.

In the first condition, the task remained identical to the practice phase, with the exception that participants were no longer informed about their performance during the final 30-second control segment.

The second condition, referred to as the segregated physical priming condition, began with a 2-second presentation of a tone sequence strongly promoting the segregated percept, followed by a 2-second silent interval, after which the standard 120-second ambiguous sequence and 30-second control segment were presented. Participants were instructed to continuously report their perception throughout. Importantly, participants were not informed in advance whether they would hear a segregated or integrated sequence.

The third condition, termed the integrated physical priming condition, was identical to the second, except that the initial 2-second priming sequence consisted of a sequence strongly promoting the integrated percept.

The fourth condition was the segregated imagery priming condition, in which participants first listened to a 30-second tone sequence strongly promoting the segregated percept, followed by a 30-second silent interval during which they were instructed to vividly imagine the segregated tone pattern to the best of their ability. This was then followed by the standard 120-second mixed sequence and 30-second control phase.

The fifth and final condition, the integrated imagery priming condition, mirrored the fourth condition, except that participants initially heard a 30-second tone sequence strongly promoting the integrated percept, then engaged in vivid mental imagery of the integrated pattern during the silent interval, before the presentation of the ambiguous sequence and control segment.

We used a counterbalanced design to control for order effects, such that participants were assigned to different condition sequences (condition 2 followed by 3, or 3 followed by 2 and condition 4 followed by 5, or 5 followed by 4). Following the priming, we recorded at 10-ms intervals whether a button was pressed and, if so, which one, with “0” indicating no response, “2” indicating a segregated percept, and “1” indicating an integrated percept.”

Table 14a Contingency table

Auditory Streams				
		Type of First Percept		
Condition		Integrated	Segregated	Total
Segregated Physical Priming	Count	59	9	68
	%	87 %	13 %	100 %
Integrated Physical Priming	Count	61	7	68
	%	90%	10%	100 %
Segregated Imagery Priming	Count	59	9	68
	%	87 %	13 %	100 %
Integrated Imagery Priming	Count	64	4	68
	%	94 %	6 %	100 %

Table 14b Chi-square analysis

	Auditory Streams				
Condition	Value	df	p	Cramer's V	BF_{10}
Physical Priming X^2	*0.283*	*1*	*0.595*	*0.046*	*0.157*
Imagery Priming X^2	*2.126*	*1*	*0.145*	*0.125*	*0.347*

Instructions

Instructions for Binocular Rivalry

The test will consist of 3 blocks, and each lasts about 5 minutes. We always calibrate at the beginning of the blocks, so you can take a break between blocks and move around, but during the experiment after calibration it is important to keep your head still.
Now I'm showing you what you're going to see, it's not part of the test yet.

Short presentation: basic rivalry + 1 imaginary block

Your two eyes both get a different view of the two monitors using the mirrors. The sinus grid moves in opposite directions. You will see the grid go in one direction and the other, and sometimes the two directions will mix. When the lanes are going in one direction, just let your eyes follow the stimulus, like watching trees go by while you're travelling. This eye movement is recorded with the eye movement tracker. Within the block, there are short sections of a few seconds, with a grey screen in between to indicate the end of the section. When this yellow dot appears, you can press enter to start the next section. So you can take a short break at this point as well, just don't move your head. But feel free to blink, you can wait a while. When this grey rectangle appears, you will have a task, which I will tell you before each block.

Now please sit here, and adjust the height to make you comfortable for the next 20 minutes or so.

Imagery Priming

In the first block, after two basic rivalry sections, you will see a grid running in one direction (both eyes will then receive the same stimulus), followed by the grey rectangle in the middle of the screen shown at the beginning. Now, for this block, when you see the grey rectangle, your task is to visualize the stimulus shown earlier (the grid running in one certain direction) onto.
Have you understood the task? Then place your chin here, rest your forehead here, look straight ahead. Before I start the calibration, find the enter with your hand, because you will be able to start the sections with it. I'm starting the calibration now, you'll see a dot, just follow it with your eyes. Okay, thank you. I'll start the test.

The first block is over, now you can move your head if you feel you need to take a break.

Physical Priming

The second block follows. Now all you have to do is follow the tracks with your eyes as if you were looking at the passing trees from a train window. We'll calibrate again. Place your chin here, rest your forehead here, look straight ahead. Before I start the calibration, find the enter with your hand. I'm starting the calibration now, you'll see the dot again, just follow it with your eyes. Okay, thank you. I'll start the test.

The second block is over, now you can move your head if you feel you need to take a break.

The third block follows. After the two basic rivalry sections, you will see a grid running clearly in one direction, followed by the grey rectangle in the middle of the screen that you know from the first block.

Conceptual Priming

For this block, after you have seen the grids going in one direction and you see the grey rectangle, your task is to say the direction that you have seen before to yourself, for example, "right, right, right" or "left, left, left."
Have you understood the task? Then place your head, look straight ahead, find the enter with your hand, and we can start the calibration. Okay, thank you. I'll start the test.

We are ready, thank you for coming!

Instructions for the Auditory Streaming Paradigm

1. Hearing Test

The hearing test lasts a few minutes. The device will first measure your left ear, then your right ear.

You will hear very soft beeping or humming sounds. You will receive a response button. Please press the button immediately when you hear a sound and keep it pressed as long as you hear it. When you no longer hear the sound, release the button and wait. If you hear it again, repeat the procedure.
As soon as you press the button, the program will start lowering the volume, so holding the button will only last a few seconds. If you feel you have been holding it for too long, briefly release it – it may only be ringing in your ear, which can happen with high tones.

Please put on the headphones so that the red mark is on your right ear.
Now I will start the program.

2. Demonstrations

During the experiment, you will hear a sequence of tones. This sequence can be perceived in different ways. If listened to for a longer time, perception may alternate between different interpretations.
Your task will be to continuously indicate what you are currently hearing.

First, volume check.

Now I will present the possible percepts so you can later report what you hear. In each demonstration, one perceptual organization will be emphasized to make it more likely that you hear that version.

Integrated Percept

First, you will hear a sequence that most likely forms a single repeating stream. This is called the integrated percept. It sounds like a "galloping" rhythm because all tones form one coherent stream. During the experiment, if you hear this percept, press and hold the assigned button (right/left).
Would you like to hear it again?

Segregated Percept

Next, the tones are arranged so they are most likely heard as two separate streams – as if a high and a low tone sequence are playing in parallel. This is called the segregated percept. I will present three versions of this percept. If you hear this during the experiment, press and hold the assigned button (left/right).
Would you like to hear it again?

In the previous example, you may have noticed that when you focus on the high tones, the low tones move to the background, and vice versa. Now we will enhance this effect. In the next example, the low-tone stream will be easier to follow, while the high tones remain in the background. If you hear this percept during the experiment, press and hold the assigned button.
Would you like to hear it again?

In the next example, the high-tone stream will be easier to follow, while the low tones remain in the background. If you hear this percept during the experiment, press and hold the assigned button.
Would you like to hear it again?

Finally, I will present the sequence used in the experiment. Just listen. This is to familiarize you with the stimulus you will hear repeatedly.
Would you like to hear it again?

3. Training

In this section, the main sequence will be played first, followed immediately (without pause) by several emphasized example sequences presented earlier.
Please continuously indicate what you hear by pressing and holding the button assigned to that percept:
If you hear the galloping (integrated) percept → press and hold its button.
If you hear the parallel (segregated) percept → press and hold its button.
If your perception switches, immediately switch to the corresponding button and hold it as long as that percept lasts.
If what you hear does not match any previously presented percept, release both buttons until one of the known percepts appears again.
There is no right or wrong answer. We are interested in your subjective perception. Perceptual switching speed varies across individuals. Do not try to force a percept – simply report what you hear.
At the end of each sequence, several emphasized example segments will follow without pause. These serve to verify that you can quickly and reliably identify the percepts.
We will repeat this practice as many times as necessary to ensure you can confidently recognize the percepts and know which button to press.

Are you ready?
You can start playback by briefly pressing either button once.

4. Experiment
Condition 1: Trials 1–3 (No Pre-cue; 3 × 1 min)
We will now begin the experiment. Each trial lasts about 1 minute. After each trial, you may take a short break.
Press and hold the button corresponding to your percept, as practiced.
After the main sequence, emphasized example segments will follow without pause to verify percept recognition.
After I start the program, you begin playback by briefly pressing either button once.

Condition 2: Trials 4–8 (Physical Pre-cue; 5 × 1 min; 1 sec pause; Segregated Pre-cue)
At the beginning of each trial, you will hear a new sound. Do not press any button – just listen.
After a very short (~1 sec) pause, the main sequence begins. Then respond as usual by pressing and holding the appropriate button.
Example segments will again follow the main sequence to verify recognition.

BREAK

Condition 1: Trials 9–11 (No Pre-cue; 3 × 1 min)
Same instructions as before: press and hold the button corresponding to your percept. Example segments follow each trial for verification. You start playback with a brief press after my signal.

Condition 3: Trials 12–16 (Passive Pre-cue; 5 × 1 min)
At the beginning of each trial, you will hear a sound previously used. Do not press any button – just listen. After a ~10-second pause, the main sequence begins.
IMPORTANT: During the pause, you have no task, simply wait for the sequence to begin.
Respond as practiced. Example segments will follow for verification.

Condition 4: Trials 17–21 (Imagery Pre-cue; 5 × 1 min)
At the beginning of each trial, you will hear a previously presented sound. Do not press any button — just listen. After a ~10-second pause, the main sequence begins.
IMPORTANT: During the pause, while waiting for the ambiguous sequence, try to imagine as vividly as possible the sound sequence presented before the pause.
Respond as practiced. Example segments will follow for verification.
